# Effects of Laser Acupuncture on Delayed Onset Muscle Soreness of the Biceps Brachii Muscle: A Randomized Controlled Trial

**DOI:** 10.1155/2019/6568976

**Published:** 2019-01-13

**Authors:** Wen-Dien Chang, Jih-Huah Wu, Nai-Jen Chang, Chia-Lun Lee, Shuya Chen

**Affiliations:** ^1^Department of Sport Performance, National Taiwan University of Sport, Taichung, Taiwan. No. 16, Section 1, Shuang-Shin Road, Taichung City 404, Taiwan; ^2^Department of Biomedical Engineering, Ming Chuan University. No. 5, De Ming Rd., Gui Shan District, Taoyuan City 333, Taiwan; ^3^Department of Sports Medicine, Kaohsiung Medical University. No. 100, Shih-Chuan 1st Road, Kaohsiung City 80708, Taiwan; ^4^Center for General Education, National Sun Yat-sen University. No. 70, Lienhai Rd., Kaohsiung City 80424, Taiwan; ^5^Department of Physical Therapy, China Medical University. No. 91, Hsueh-Shih Road, Taichung City 404, Taiwan

## Abstract

**Objectives:**

The aim of this study was to explore the effects of laser acupuncture on improvement of recovery and muscle performance in delayed muscle soreness (DOMS) when applied before exercise.

**Methods:**

This randomized, blinded, and controlled study included healthy participants (n = 40) who were randomized into laser acupuncture and placebo groups. Laser acupuncture was applied to the Tianquan (PC2) and Chihtseh acupoints (LU5) at a dose of 36 J and energy density of 9.7 J/cm^2^ before inducing DOMS. The placebo group received sham laser acupuncture with no laser output. Visual analog scale (VAS), proprioception, pressure pain threshold (PPT), arm circumference, and muscle strength were observed at the baseline and 24, 48, 72, and 96 h after induction of DOMS.

**Results:**

Significant changes in the VAS (F_4,  43.96_ = 31.47;* p* = 0.001), PPT (F_4,  1.35_ = 35.07;* p* = 0.001), normalized arm circumference (F_4,  0.001_ = 3.87;* p* = 0.005), and normalized muscle strength (F_4,  0.31_ = 24.99;* p* = 0.001) were observed within the groups over time (*p* < 0.05), but there were no significant differences between the two groups (*p* > 0.05). Normalized arm circumference was significantly different between the two groups at 48 and 72 h after induction of DOMS (*p* < 0.05).

**Conclusion:**

Photobiomodulation therapy on Tianquan (PC2) and Chihtseh acupoints (LU5) before the exercise did not significantly decrease DOMS and increase muscle performance. Laser acupuncture as a supplemental therapy seemed to have no effect on DOMS prevention.

## 1. Introduction

Delayed muscle soreness (DOMS) is observed after sport in elite athletes, and it may also affect the general population [1]. It is a myogenic condition often caused 24 to 72 h after performing exercises that overload the muscles. DOMS is not inflammatory in nature [1]. The symptoms of DOMS, which include pain, muscle tenderness, and loss of joint range of motions, are alleviated over time [2]. However, accompanying symptoms, such as muscular discomfort, often affect the sports performance and training schedule of the athlete [2]. The previous study of Cheung et al. revealed that DOMS could increase the risk of injuries when the affected individual returns to the sport after recovery [3]. Therefore, the development of effective preventions that can help prevent DOMS may prove beneficial for athletes.

Acupuncture is a supplemental therapy in traditional Chinese medicine. It involves the use of needles that are placed on acupuncture points, which alters the qi and blood circulation along the meridians, thus exerting its therapeutic effects [4]. Acupuncture points commonly are located in a tender point or muscular pain site, and qi occurs to transmit energy through meridians by acupuncture [5]. The study of Hübscher et al. reported that acupuncture for DOMS resulted in reducing pain and increasing muscle strength when applied on acupuncture points of Yanglingquan (GB34), Tianfu (LU3), Chize (LU5), Quchi (LI11), Xuehai (SP10), and ah-shi (tender points) [6]. The needle acupuncture is manipulated to create a de-qi sensation, and participants could feel soreness, numbness, heaviness, and distension around the acupuncture point [5]. It could activate the meridian, but the effects of acupuncture vary significantly based on the physician's technology [7]. Based on the theory of traditional Chinese medicine for musculoskeletal diseases, disharmony between ying and wei causes muscle soreness and tightness in DOMS. In clinic practice, the signs of asthenia in renal yin and yang and loss of renal qi can be treated with acupuncture by focusing on the pericardium meridians of hand-Jueyin and hand-Shaoyin. The acupuncture points, that is, Tianquan (PC2) and Chihtseh (LU5) acupoints, were chosen as they corresponded with the injured areas on the biceps muscle tendon. Through transmitting qi through the specific meridians, the active energy may improve the muscle fatigue recovery. It could be an advanced treatment that acupuncture also may be used before exercise to enhance the muscle recovery ability and prevent sports injury.

Fleckenstein et al. indicated that acupuncture was not effective in the treatment of symptoms after inducing DOMS [8]. The biases of physicians' technique and non-quantifiable dose could affect the effects of acupuncture. Laser acupuncture, which uses low-level lasers on the acupuncture points to activate the meridians, has several advantages such as noninvasiveness and safety and the ability to quantify the dose and apply the same amount repeatedly in order to achieve the desired therapeutic effect [9]. The systematic review of Law et al. provided moderate evidence of the use of laser acupuncture for the management of musculoskeletal pain, using an appropriate treatment dosage [10]. Laser acupuncture could provide quantifiable dosages to stimulate acupuncture points and to explore the analgesic effect as a result of the laser biostimulation [9]. However, there is a dearth of studies exploring the effect of laser acupuncture on DOMS. Laser biostimulation is also called photobiomodulation and could be used on injured muscle tissue. Laser light could irradiate on muscle tissue to increase mitochondrial activity. Cytochrome c oxidase absorbed photons to increase adenosine triphosphate and provide energy source for muscle tissue recovery. Lopes-Martins et al. used the output power of 2.5 mW and wavelength of 655 nm on fatigued tibial anterior muscle. It was the first study of laser biostimulation in rats and was used to prove the effect on decreasing muscle damage after high intense exercise in animal study [11]. The studies of Leal Junior et al. are the first randomized controlled trials to investigative the effects of photobiomodulation on DOMS [12, 13].

Some systematic review studies indicated preexercise photobiomodulation could provide ergogenic effect on recovery and muscle performance of DOMS [14, 15]. The evidences on preexercise photobiomodulation to prevent muscle injury and improve recovery were proved. The development of supplemental therapies for DOMS prevention has also shown an increasing trend over the past few decades. Laser acupuncture is a quantitative treatment, and also have advantages of safety and convenient. The use of laser acupuncture before the development of DOMS may prove beneficial for the prevention of sports injuries and provide additional protection during exercise. Some studies have found that the use of photobiomodulation therapy on muscles with DOMS before exercise could reduce pain and decrease creatine kinase levels in athletes [16–19]. However, to the best of our knowledge, no studies have been conducted on the effect of laser acupuncture before exercise, and its effects on decrease of DOMS and increase of muscle performance. Therefore, the aim of the present study was to explore the effects of laser acupuncture before the induction of DOMS, and changes in the visual analog scale (VAS), proprioception, pressure pain threshold (PPT), arm circumference, and muscle strength were examined 24, 48, 72, and 96 h after DOMS induction.

## 2. Methods

This randomized and triple blinded controlled trial was approved by the Institutional Review Board of China Medical University and Hospital (No. CMUH106-REC1-090). Informed consent was obtained from the participants in this study. Based on the study by Hübscher et al. [6], a significant difference of 1.6 in VAS was detected, and statistical power of 80% and *α* level of 0.05 were calculated. The sample size was estimated at 20 in each group by G*∗*Power software (version 3.1.9.2; Heinrich-Heine-Universität, Düsseldorf, Germany). The participants in the present study comprised healthy college students belonging to the sports team at Ming Chuan University, Taiwan. Healthy individuals were included in this study, whereas the exclusion criteria included the following: presence of muscle soreness and tenderness, acupuncture within a period of 1 week, and the use of any drugs or medication for musculoskeletal conditions. Each participant completed six visits. The first visit was used to collect demographic data, and baseline assessments were performed in both groups before the laser acupuncture. Subsequent visits were used to assess the VAS, proprioception, PPT, arm circumference, and muscle strength, in order to ascertain the changes in DOMS symptoms 24, 48, 72, and 96 h after the induction of DOMS procedure.

The recruited participants were divided into laser acupuncture and placebo groups. A simple drawing of lots (A or B) was used to determine the grouping, and an assistant, who was not involved in this experiment, handled the randomization procedure. The laser instruments were classified as active (A lot) and nonlaser (B lot) output modes, and the appearance and mode of operation were similar. The laser instruments were applied by one therapist. The exercise for inducing DOMS and subsequent assessments were implemented by an athletic trainer. The outcome data were calculated and analyzed by an analyst. The participants, physical therapist, technician, and analyst were all blinded to the type of laser instrument used.

### 2.1. Laser Acupuncture

Laser acupuncture was performed before DOMS induction using the laser instrument, Painless Light PL-830 (Advanced Chips & Products Crop., USA) at an output frequency of 10 Hz, a wavelength of 830 nm, a total output power of 60 mW, a dose of 36 J, and energy density of 9.7 J/cm^2^ (Table 1). The laser was irradiated directly onto the Tianquan acupoint (PC2) and Chihtseh acupoint (LU5) on each arm. Tianquan acupoint (PC2) is located between the short and long heads of the proximal humeral biceps muscle, and it is an acupoint for the pericardium meridian of hand-Jueyin. Chihtseh acupoint (LU5) is located at the distal tendon of the humeral biceps muscle, and it is an acupoint for the heart meridian of hand-Shaoyin [20]. Each acupuncture point was irradiated for 10 min. Participants in the placebo group underwent the same procedures as those in the laser acupuncture group, but the laser instrument was placed on the points without any laser output.

### 2.2. Induction Procedure

A dumbbell was used for free-weight training to induce DOMS in the nondominant arm. Prior to induction, the participants were asked to perform one round of the elbow-flexor stretching exercise for 60 s. The recruited participants were seated on a stable chair, and one repetition maximum in elbow flexion was determined as the maximum amount of muscle force. One repetition maximum of the elbow flexors is determined by lifting dumbbell in 0.5 kg increments, and the participants were encouraged to elicit the maximal weight. The weight of the dumbbell, at 75% one repetition maximum, was used to perform the eccentric muscle contraction exercise of the elbow flexor muscles. The participant was instructed to lift the weight and put it down as slowly as possible. Verbal encouragement was used to urge them to perform as many continuous exercises as possible. Repeated exercise procedures with 30 s rest intervals were between the procedures. The procedure continued until participants' subjective muscle exhaustion [21], and the period of exhaustive exercise was assessed and checked by the physical therapist.

### 2.3. Outcome Measures

The outcome assessments, including VAS, proprioception, pressure pain threshold, arm circumference, and muscle strength were evaluated by the same physical therapist, who had 10 years of clinical experience. The extractive data of outcome were blinded to be analyzed by another analyst.

### 2.4. Visual Analog Scale

Pain intensity of the biceps muscle during manual resisted isomeric test was evaluated using a VAS. The VAS involved a 10 cm horizontal line used to assess the intensity of muscle soreness. It determined the pain intensity and had high reliability (intraclass correlation coefficients = 0.97) [22]. The participants were seated with the elbow flexed at 90° and were instructed to perform an isometric elbow flexor contraction for 5 s and score the intensity of muscle soreness on the scale (0 cm “no pain” and 10 cm “extreme soreness”) [6].

### 2.5. Proprioception

Force sense is one kind of proprioception and is a high-reliable measure. At first, each participant was seated with the elbow in flexion at 90° and performed maximum voluntary isometric contraction against a handheld dynamometer (MicroFET3; Hoggan Health Industries Co., UT, USA). The elbow flexor muscles at 50% maximal voluntary isometric contraction for 3 s was determined as the target force [23]. This valid assessment is a force sense testing and has moderate test-retest reliability (intraclass correlation coefficients = 0.70) [23]. A handheld dynamometer was used to perform the exercises several times with visual feedback and then with 1 min rest intervals. The measurements were repeated three times, and the average of three trials (absolute error value) was calculated.

### 2.6. Pressure Pain Threshold

The minimum amount of force required to induce muscle pain was assessed by measuring the pressure pain threshold (PPT) using a pressure algometer (Wagner Pain Test™ Model FPK 40; Wagner Instrument, Greenwich, USA). The upper arm was marked with eight equidistant points at 4 cm intervals along the median line of the biceps muscle from the bicipital groove to the radial insertion. Manual pressure at a constant speed of 10 N/s was applied to the points through the head (diameter, 1 cm) of the pressure algometer, and it was stopped when the participant began to feel muscle tenderness [24]. The muscle tenderness is a valid and clinical physical evaluation for assessing the subjective muscle pain and has moderate test-retest reliability (intraclass correlation coefficients = 0.77) for the elbow region [25]. The average PPT of the eight assessed points was calculated for statistical analysis.

### 2.7. Arm Circumference

The participants were seated with their arms resting beside their trunks. Based on the eight equidistant points used to measure the PPT, a tape measure was used to measure the circumference of the arm at the point and was wrapped tightly around the bicep, and the measurement was recorded. The average value of each measured point was calculated. This assessment was valid for upper extremity edema of DOMS and had high reliability (intraclass correlation coefficients = 0.95) [26].

### 2.8. Muscle Strength

The isometric strength of the elbow flexor muscles was assessed using a digital muscle testing dynamometer (MicroFET3, Hoggan Health Industries Co., Utah, USA). The dynamometer was placed at the wrist to measure perpendicular force during the movement proceeds toward elbow flexion. The participant was seated with the elbow in flexion (90°), and the dynamometer sensor was contacted on 1 inch above styloid process of ulna on flexor surface. They were asked to perform maximal voluntary isometric contractions for 5 s [27]. The procedure was repeated three times with 1-minute rest intervals. This assessment had moderate test-retest reliability (intraclass correlation coefficients = 0.77) for elbow flexor muscle and was valid for muscle strength [28].

## 3. Statistical Analysis

Data were analyzed using SPSS Version 17 (SPSS Inc., Chicago, IL, USA). Descriptive statistics of the participants' demographic data, including age, height, weight, and body mass index, and baseline assessments of both groups, are presented as mean ± standard deviation. An independent t-test was used to compare the assessed variables between the two groups. Proprioception, arm circumference, and muscle strength were normalized against the baseline assessment values obtained before inducing DOMS. Two-way mixed analysis of variance (2 groups × 5 times) with repeated measures over time was used to compare the groups. Mauchly's sphericity test was used to validate the conditions and assumptions of applying the repeated-measures test. The Greenhouse–Geisser test was performed if the sphericity assumption did not hold, and the Bonferroni correction was used for multiple comparisons. The assessed variables are presented as mean ± standard error. A significance level of* p* < 0.05 was set for all the analyses.

## 4. Results

**Figure 1:**
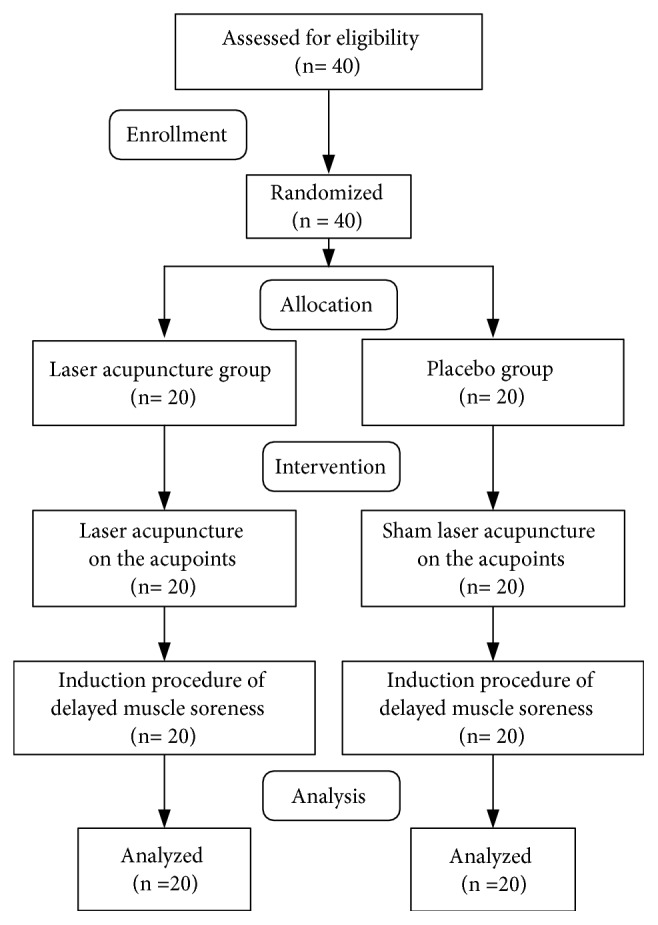
Flow chart of the present study.

Forty participants were included (Figure 1) and were randomly divided into a laser acupuncture group (n = 20) and a placebo group (n = 20). All participants completed the study procedure with no dropouts and no adverse reactions reported. No significant differences in age, height, weight, or body mass index were observed between the two groups (*p* > 0.05; Table 2). Furthermore, no significant differences in baseline assessment values of force sense, arm circumference, or muscle strength (*p* > 0.05; Table 2) were noted between the two groups. All participants did not present with muscle soreness or tenderness before DOMS induction. Hence, the VAS was 0, and the upper limit PPT was set at 5 kg/cm^2^ for avoiding tissue bruising.

**Figure 2:**
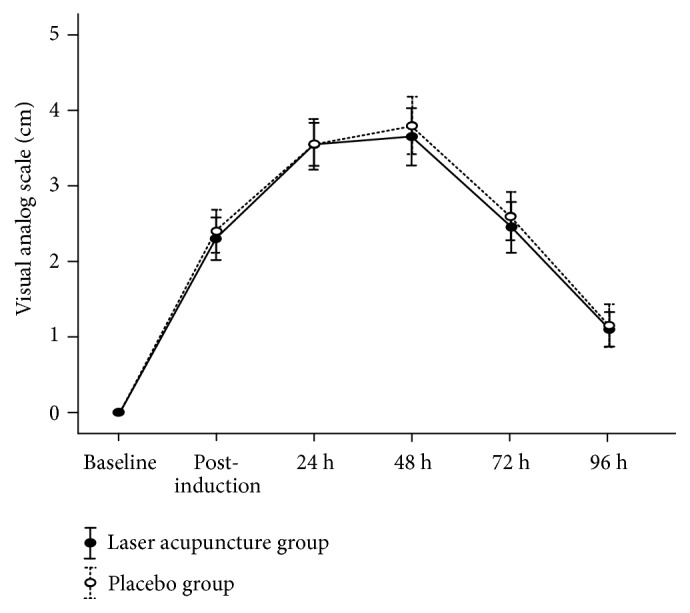
Visual analog scale (mean ± standard error) of muscle soreness from 2 groups at baseline, postinduction, and 24, 48, 72, and 96 h after inducing delayed muscle soreness.

However, after induction of DOMS, the VAS was increased in both groups, with no significant differences observed between the two in subsequent assessments (*p* > 0.05; Figure 2 and Table 3). Maximum pain occurred at 48 h in both groups. A significant main effect for time (F_4,  43.96_ = 31.47;* p* = 0.001) was observed, but no significant main effects were noted for the group (F_1,  0.41_ = 0.09;* p* = 0.76) or group × time interactions (F_4,  0.43_ = 0.03;* p* = 0.99).

**Figure 3:**
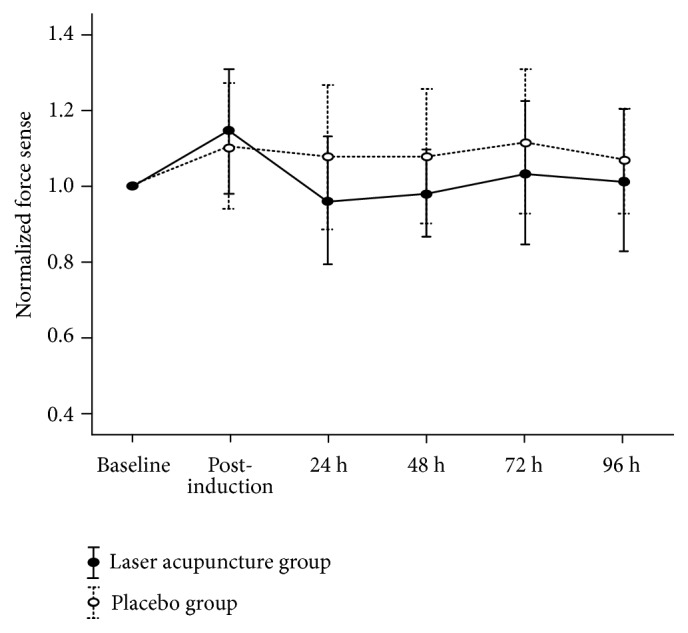
Normalized force sense (mean ± standard error) from 2 groups at baseline, postinduction, and 24, 48, 72, and 96 h after inducing delayed muscle soreness.

No significant main effects on normalized force sense were noted for the group (F_1,  0.17_ = 0.13;* p* = 0.71), time (F_4,  0.72_ = 0.18;* p* = 0.94), or group × time (F_4,  0.39_ = 0.10;* p* = 0.98). In both groups, normalized force sense was increased after the induction of DOMS, but no significant differences in values were observed in subsequent assessments (*p* > 0.05; Figure 3 and Table 3). The lowest normalized force sense was measured at 24 h, with the laser acupuncture group presenting lower 0.12 value of normalized force sense, when compared with the placebo group.

**Figure 4:**
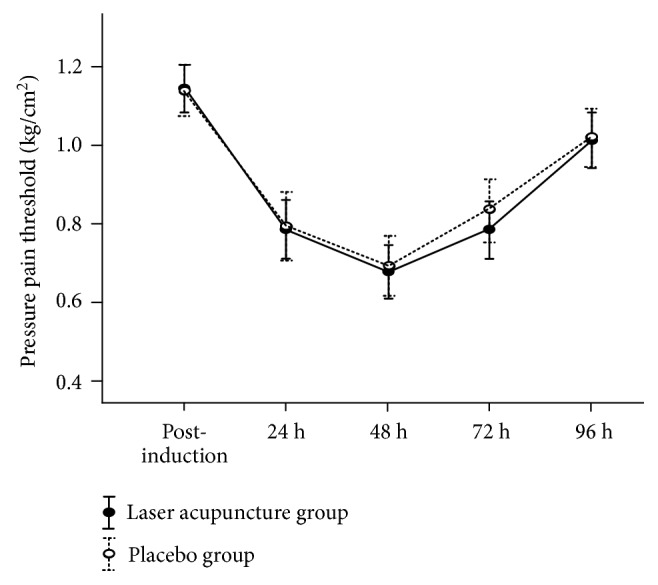
PPT (mean ± standard error) from 2 groups at postinduction and 24, 48, 72, and 96 h after inducing delayed muscle soreness.

A significant main effect on PPT for time (F_4,  1.35_ = 35.07;* p* = 0.001) was observed, but not for group (F_1,  0.01_ = 0.29;* p* = 0.86) or group × time interactions (F_4,  0.01_ = 0.11;* p* = 0.98). Similar to the VAS, maximum PPT occurred 48 h after DOMS induction. In Figure 4 and Table 3, the laser acupuncture group exhibited lower 0.02 kg/m^2^ of PPT when compared with the placebo group.

**Figure 5:**
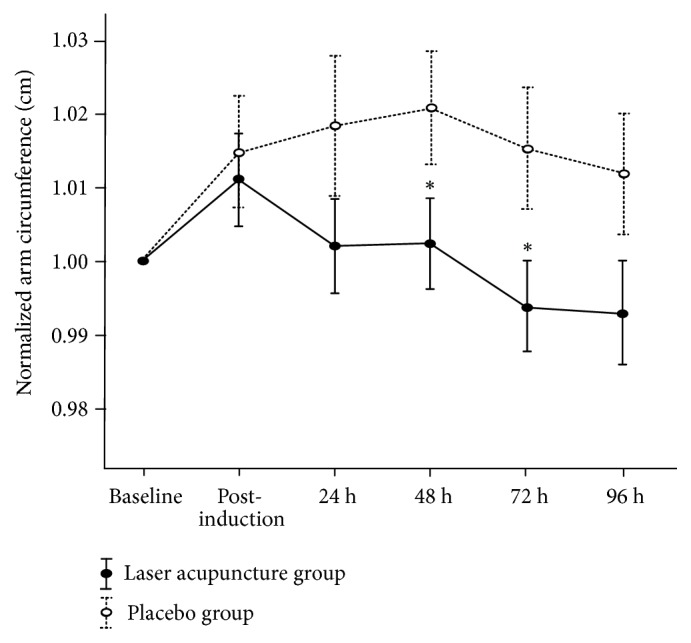
Normalized arm circumference (mean ± standard error) from 2 groups at baseline, postinduction, and 24, 48, 72, and 96 h after inducing delayed muscle soreness. *∗P* < 0.05, significant difference between groups.

No significant main effects on normalized arm circumference for group (F_1,  0.01_ = 1.79;* p* = 0.18) or group × time interactions (F_4,  0.001_ = 2.04;* p* = 0.09) were noted. Nevertheless, a significant main effect for time (F_4,  0.001_ = 3.87;* p* = 0.005) was observed. In addition, normalized arm circumference was highest at 48 h after DOMS induction, with significant differences at 48 and 72 h between the two groups (*p* = 0.04; Figure 5 and Table 3).

**Figure 6:**
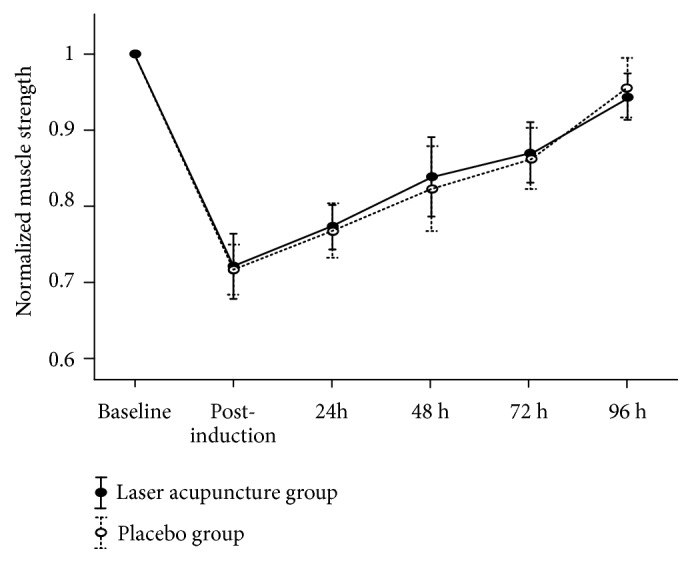
Normalized muscle strength (mean ± standard error) from 2 groups at baseline, postinduction, and 24, 48, 72, and 96 h after inducing delayed muscle soreness.

Similar to the arm circumference measurement values, no significant main effects on normalized muscle strength for group (F_1,  0.001_ = 0.01;* p* = 0.93) or group × time interactions (F_4,  0.001_ = 0.07;* p* = 0.98) were observed (Figure 6 and Table 3). A significant main effect for time (F_4,  0.31_ = 24.99;* p* = 0.001) was found. Normalized muscle strength demonstrated a gradual increase during the subsequent assessments, but no significant differences were observed between the two groups.

## 5. Discussion

The present study was focused on clinical outcomes, that is, VAS, proprioception (force sense), PPT, arm circumference, and muscle strength, and used low-level laser on the acupuncture points before DOMS induction. In both groups, decreased VAS and PPT occurred at 48 h after DOMS induction, and increased normalized muscle strength occurred at postinduction. But, there were no significant differences on VAS, PPT, normalized force sense, and normalized muscle strength at all assessment times between both groups. Only arm circumference was significantly decreased 48–72 h after induction in the laser group when compared with the sham laser group.

Traditional acupuncture involves the application of needles on acupuncture points for mechanical stimulation. By contrast, laser acupuncture causes photobiomodulation to activate neurophysiological pathways for analgesia (inhibition of spinal and supraspinal descending pathways) and modulate neurotransmitters [29]. Fleckenstein et al. were the first to compare the effects of laser acupuncture with verum acupuncture after the induction of DOMS [8]. Their results showed that laser acupuncture had no effects on the treatment of DOMS, because this treatment cannot penetrate into local deep muscle tissue. However, they indicated that the treatment mechanisms involved in traditional and laser acupuncture were presumed to be similar.

Leal Junior et al. are pioneers in this field and their series of studies had provided positive evidences on low-level laser for DOMS. Both low-level laser parameters (655 nm wavelength, 5 J; energy density of 500 J/cm^2^; 830 nm wavelength, energy density of 500 J/cm^2^) could decrease the increase of blood lactate level [12, 13]. In another study, low-level laser parameter (energy density: 1,428.57 J/cm^2^) could inhibit the increase of CK level and acceleration of lactate removal in athletes. These findings provided the basis for other studies [30]. Baroni et al. had applied low-level laser on quadriceps muscle before knee extensor eccentric exercise and then lowered lactate dehydrogenase and serum creatine kinase levels and enhanced the recovery of the damaged muscles which were found in biochemical analysis [17].

Hübscher et al. evaluated the effects of acupuncture on Chihtseh (LU5) acupoint and other accompanied acupoints in delayed-onset muscle soreness after exercise, and they reported an improvement in both the VAS and PPT after 48–72 h [6]. Barlas et al. also indicated that acupuncture on Chihtseh (LU5) acupoint and other accompanied acupoints exerted analgesic effects until 120 h, which were significantly higher than those in the placebo and control groups [31]. Unlike previous studies on the effect of acupuncture treatment on DOMS, our results of the present study indicated that laser acupuncture on Tianquan (PC2) and Chihtseh (LU5) acupoints before the induction of DOMS had no significant decrease in VAS and PPT to compare with control group. To the best of our knowledge, there are no reports on the effects of acupuncture or laser acupuncture before DOMS induction. It seemed that laser acupuncture had no effects on pain relief for prevention of DOMS occurring. Further studies evaluating the use of the appropriate acupuncture treatment method on various acupoints to prevent the occurrence of DOMS were suggested to explore.

Photobiomodulation therapy was used as the treatment tool. The therapeutic effects were dependent on laser irradiation parameters such as the applied wavelength, dosage, and pulses. Very high or very low values of the parameters could influence the treatment effects of the laser, which is “Arndt-Shultz law” [32]. Dos Reis et al. applied photobiomodulation therapy (830 nm) on quadriceps muscles before and after exercise, and they reported reduced levels of serum lactate and creatine kinase before the induction of DOMS [33]. In the systematic review study with meta-analysis, Vanin et al. investigated photobiomodulation therapy on the improvement of muscular performance and reduction of muscular fatigue. Their positive results were the wavelength (655-950 nm), energy dose (60-300 J), and maximal power output per diode (200 mW) [34]. In the present study, 830 nm laser acupuncture at a total energy of 9.7 J/cm^2^ and total energy of 36J was tried before DOMS induction. The pain and discomfort of DOMS successfully presented with at 48 h. Comparing with the placebo group, the pain was not lowered significantly during the subsequent assessment times in the laser acupuncture group. Insufficient energy of photobiomodulation therapy may be the source of problem in this study. Application of photobiomodulation therapy on muscle could increase the mitochondrial respiratory rate and adenosine triphosphate synthesis, thereby improving cellular metabolism and decreasing the accumulation of pain factors before muscle fatigue [8]. In the current study, the energy of the laser acupuncture was directed on the acupuncture points, but not on the affected muscles belly. Therefore, the local laser acupuncture could not exert significant bioenergetic effects at the cellular level. Laser acupuncture as a supplemental therapy for DOMS prevention may cause no effects on reducing the symptoms.

Athletes need to return to sports approximately 48 h after high intensity training, which is often accompanied with DOMS. Thus, the recovery of muscle performance including muscle strength and proprioception is essential [35]. Antonialli et al. used photobiomodulation therapy with doses of 10, 30, and 50 J on quadriceps muscle before the eccentric contraction protocol [36]. They found that 30 J dose significantly increased maximum voluntary contraction compared to placebo group. It seems that 30 J dose, 905 nm wavelength of laser pulsed 640 nm wavelength of red LEDs, and irradiated site (6 locations in muscle belly of quadriceps) represented an effect on increased muscle strength. However, in the current study, we found that 36 J dose and 830 nm wavelength of photobiomodulation on Tianquan (PC2) and Chihtseh acupoints (LU5) could not recover the muscle performance. Borges et al. indicated that photobiomodulation therapy applied immediately on the muscle after eccentric contraction exercise could promote the increase in muscle strength and improve the recovery time of the fatigued muscle [37]. During occurring muscle fatigue, loss in muscle strength was also accompanied by a disturbance in muscular proprioception [38]. Mesquita-Ferrari et al. confirmed that photobiomodulation therapy could decrease tumor necrosis factor-alpha (TNF-*α*) by modulating the expression of the cytokine [39]. TNF-*α* is a proinflammatory cytokine and can affect the muscle contraction force [40]. Therefore, photobiomodulation therapy could decrease muscle strength loss in DOMS. The results of the present study indicate that laser acupuncture did not provide better muscle strength recovery at 48 h, and the difference was not significant when compared with the sham laser acupuncture group. This may be attributed to the same reason that the laser acupuncture was not applied to the affected muscles, resulting in nonsignificant changes. In a previous animal study, Onda et al. noted that acupuncture could modulate messenger RNA expression levels of atrogin-1 and muscle RING-finger protein-1 (MuRF1) [41], leading to an increase in protein synthesis and decrease in degradation, thereby preventing skeletal muscle atrophy and muscle strength loss. Nonetheless, this mechanism was not proved in human study. A similar tendency was observed in the present study, wherein the outcome of muscle strength and proprioception in the laser acupuncture group was not better than that in the placebo group, and the difference was not statistically significant.

The arm circumference is increased due to exercise-induced muscle damage. The DOMS occurs because of structural damage to the micro contractile filaments in the muscle, resulting in pain and edema [42]. In the present study, the differences in arm circumference were significantly presented at 48 and 72 h between two groups, and this means that laser acupuncture could improve the recovery of edema. Lorenzini et al. indicated that laser acupuncture had an antiedema effect in an animal model of complete Freund's adjuvant-induced inflammatory pain [43]. This effect of laser acupuncture was also found in the subcutaneous tissues of osteoarthritic patients by sonographic examination [44]. The evidences of a decrease in reactive oxygen species [45] and improvements in antioxidant capacity and adenosine triphosphate synthesis also support the effect of low-level laser for DOMS [46]. However, the change of arm circumference is only one finding that supports that laser acupuncture before DOMS induction could decrease edema in the injured muscle. But, the physiological mechanism of laser acupuncture is still not clear to confirm this finding.

There are some limitations in the present study. First, the study design should have a nontreated group as a control group to investigate the psychological effect. Second, appropriate laser parameters and preventive acupuncture points on DOMS were still unclear. Usage of various laser acupuncture parameters and differential acupoints were suggested in future studies.

## 6. Practical Relevance

Photobiomodulation therapy on Tianquan (PC2) and Chihtseh acupoints (LU5) before the exercise cannot decrease DOMS and increase muscle performance. It seems that muscles may be preconditioned by photobiomodulation to improve the reduction of muscle edema.

## 7. Conclusion

Laser acupuncture applied on Tianquan (PC2) and Chihtseh (LU5) acupoints before the induction of DOMS significantly decreased the arm circumference at 48 and 72 h.

But it seemed to have no effects of the DOMS prevention on pain, proprioception, PPT, and muscle strength. Laser acupuncture as a supplemental therapy to reduce DOMS symptoms should be probed in future studies.

## Figures and Tables

**Table 1:** The instrument parameters of photobiomodulation therapy.

Parameters	Value
Laser mode	830 nm
Pulse frequency	10 Hz
Pulse width	0.75 ms
Peak power	8 W
Treatment time per point	10 min
Energy	60 mW
Energy density	9.7 J/cm^2^
Number of treated points	2 acupoints
Total energy	36 J

**Table 2:** Demographic characteristics of the recruited participants.

	Laser acupuncture group	Placebo group	*P* value
	(n=20)	(n=20)	
Age (years)	20.90±1.12	21.05±1.53	0.72
Height (cm)	165.96±7.34	164.56±6.56	0.53
Weight (kg)	59.62±7.87	61.23±6.91	0.49
Body mass index (kg/m^2^)	21.74±3.38	22.69±3.05	0.35
VAS	0	0	1
Force sense (Ib)	1.34±0.84	1.31±1.01	0.92
PPT (kg/cm^2^)	5	5	1
Arm circumference (cm)	29.02±3.65	29.18±3.46	0.89
Muscle strength (Ib)	47.62±14.22	47.83±13.54	0.96

VAS, visual analog scale; PPT, pressure pain threshold.

**Table 3:** Outcomes at baseline, postinduction, and 24, 48, 72, and 96 h after inducing delayed muscle soreness.

Items	Groups	Postinduction	24 h	48 h	72 h	96 h
VAS	Laser acupuncture group	2.28±1.07	3.51±1.52	3.67±1.01	2.43±1.43	1.09±0.84
	Placebo group	2.37±0.94	3.53±1.01	3.81±1.65	2.56±1.83	1.21±0.58
Normalized force sense	Laser acupuncture group	1.15±0.76	0.94±0.71	0.98±0.54	1.03±0.84	1.01±0.08
	Placebo group	1.11±0.80	1.07±0.84	1.07±0.80	1.11±0.89	1.06±0.62
PPT	Laser acupuncture group	1.15±0.31	0.78±0.36	0.67±0.27	0.78±0.31	1.01±0.37
	Placebo group	1.14±0.37	0.79±0.31	0.69±0.37	0.84±0.42	1.02±0.30
Normalized arm circumference	Laser acupuncture group	1.01±0.02	1.02±0.03	1.00±0.03	0.99±0.02	0.99±0.03
	Placebo group	1.01±0.03	1.00±0.04	1.02±0.02*∗*	1.02±0.04*∗*	1.01±0.03
Normalized muscle strength	Laser acupuncture group	0.72±0.78	0.77±0.13	0.83±0.22	0.87±0.17	0.94±0.31
	Placebo group	0.71±0.13	0.74±0.09	0.82±0.22	0.86±0.13	0.95±0.17

*∗p*<0.05, laser acupuncture group versus placebo group

## Data Availability

The data used to support the findings of this study are included within the article.
